# Hypoxic–ischemic injury causes functional and structural neurovascular degeneration in the juvenile mouse retina

**DOI:** 10.1038/s41598-021-90447-5

**Published:** 2021-06-16

**Authors:** Ismail S. Zaitoun, Pawan K. Shahi, Andrew Suscha, Kore Chan, Gillian J. McLellan, Bikash R. Pattnaik, Christine M. Sorenson, Nader Sheibani

**Affiliations:** 1 Department of Ophthalmology and Visual Sciences, University of Wisconsin School of Medicine and Public Health, 1111 Highland Avenue, WIMR 9418, Madison, WI 53705 USA; 2 McPherson Eye Research, University of Wisconsin School of Medicine and Public Health, Madison, WI USA; 3 Department of Pediatrics, University of Wisconsin School of Medicine and Public Health, Madison, WI USA; 4 Department of Surgical Sciences, School of Veterinary Medicine, University of Wisconsin, Madison, WI USA; 5 Department of Cell and Regenerative Biology, University of Wisconsin School of Medicine and Public Health, Madison, WI USA

**Keywords:** Cell biology, Developmental biology, Molecular biology, Neuroscience

## Abstract

Ischemic stroke is a major cause of long-term disabilities, including vision loss. Neuronal and blood vessel maturation can affect the susceptibility of and outcome after ischemic stroke. Although we recently reported that exposure of neonatal mice to hypoxia–ischemia (HI) severely compromises the integrity of the retinal neurovasculature, it is not known whether juvenile mice are similarly impacted. Here we examined the effect of HI injury in juvenile mice on retinal structure and function, in particular the susceptibility of retinal neurons and blood vessels to HI damage. Our studies demonstrated that the retina suffered from functional and structural injuries, including reduced b-wave, thinning of the inner retinal layers, macroglial remodeling, and deterioration of the vasculature. The degeneration of the retinal vasculature associated with HI resulted in a significant decrease in the numbers of pericytes and endothelial cells as well as an increase in capillary loss. Taken together, these findings suggest a need for juveniles suffering from ischemic stroke to be monitored for changes in retinal functional and structural integrity. Thus, there is an emergent need for developing therapeutic approaches to prevent and reverse retinal neurovascular dysfunction with exposure to ischemic stroke.

## Introduction

Ischemic stroke is a significant cause of long-term disabilities and death as a result of permanent or transient disruption of the regular blood supply to the brain and/or retina^1^. Typically, ischemic stroke occurs in older individuals but can occur at a lower rate in the pediatric population (1 month to 18 years of age)^2,3^. The ischemic stroke incidence in the pediatric population is around 2 in 100,000^4–8^. Numerous risk factors contribute to the occurrence of childhood ischemic stroke with arteriopathies, the primary cause in children^9,10^. Arteriopathy is a group of vascular diseases including focal or transient cerebral arteriopathy^11,12^, craniocervical arterial dissection^13–17^, fibromuscular dysplasia^18^, Moyamoya disease^19–21^ and primary CNS angiitis^22^. Some non-arteriopathy congenital vascular syndromes such as Sturge–Weber^23^, incontinentia pigmenti^24^, and PHACE syndrome^25^ are also associated with childhood ischemic stroke. Additionally, children with cardiac diseases^26–29^, inherited or acquired thrombophilia diseases^30–37^, sickle cell disease^38,39^, cancers^40–42^, inborn errors of metabolism (i.e., mitochondrial disorders and Fabry Disease)^43,44^, and autoimmune diseases (i.e., systemic vasculitis and systemic lupus erythematosus)^45,46^ have an increased incidence of ischemic stroke. Most of these risk factors are associated with ocular manifestations such as central retinal vein occlusion, hemorrhage, avascularity, or neovascularization.

Retina has the highest oxygen consumption rate per tissue volume as compared to other organs, including brain^47–49^. In neonates and adults, the retina is sensitive to ischemia/hypoxia conditions^50,51^. It is estimated that ~ 60% of adults with acute stroke have visual impairments^52^. However, data regarding the prevalence of visual impairments after ischemic stroke in the pediatric population is lacking^50^. Although retinal ischemia and reperfusion in adult mice damage both the retinal neurons and the vasculature^53^, there is a lack of evidence to the outcome of ischemia in younger juvenile mice. We recently reported the attenuation of retinal neurovascular development in neonatal mice after exposure to hypoxic–ischemic (HI) conditions^54^. Here we determined the impact of HI on the retinal neurovascular integrity of 30-days old mice, equivalent to juvenile age in humans. We found that the functional and structural integrity of the neuroretina was impaired as well as retinal blood vessels degenerated after HI. Thus, HI has a profound impact on the juvenile mouse retina.

## Materials and methods

### Ethics statement and animals

Experiments were performed in accordance with the National Institutes of Health Guide for the Care and Use of Laboratory Animals and approved by the Institutional Animal Care and Use Committee of the University of Wisconsin School of Medicine and Public Health. Studies were conducted in compliance with the ARRIVE guidelines. C57BL/6J mice were obtained from the Jackson Laboratory. Mice were allowed ad libitum access to standard rodent chow and water. The day of the birth was considered postnatal day zero (P0). Both male and female mice were used in this study. Animals from both sexes were randomly assigned to three independent cohorts. Single cell counting was conducted by an investigator blinded to the control and injured eye groups. Animals were euthanized using either inhalation of isoflurane or carbon dioxide.

### HI induction in juvenile mice

Hypoxia and ischemia were induced in postnatal day 30 (P30) C57BL/6J mice. P30 is considered a juvenile stage of mouse development, during which the vascular and the nervous systems are reaching maturity. The animals were anesthetized with isoflurane (Butler Schein Animal Health Supply, Reno, NV) (5% for induction, 2–3% for maintenance) in 30% oxygen mixed with nitrous oxide. The body temperature of the mice was maintained at 36 °C using a heated surgical table. Under a surgical microscope, a midline skin incision was made in the ventral neck, and the trachea was visualized through the muscle overlying it. The left common carotid artery was freed from the left common jugular vein and vagus nerve by blunt dissection, electrically cauterized and cut. In addition to the brain, the hypoxic–ischemic insults generated by this procedure apply to the eye as well; the ophthalmic artery is a branch from the common carotid artery. The incision was injected with 0.5% bupivacaine and closed with a single 6-0 silk suture. Animals were returned to their cages and monitored continuously for a 2 h recovery period. To induce unilateral ischemic injury, animals were then placed after 2 h of the left common artery occlusion in a hypoxia chamber (BioSpherix Ltd, Redfield, NY) equilibrated with 10% O_2_ and 90% N_2_ at 36 °C for 50 min. This is a well-characterized model of neonatal HI and results in reproducible brain injury ipsilateral to the electrocauterized and transected left common carotid artery. We have successfully used this model to study the effect of HI on the neurovascular retina in mouse neonates^54^.

### Full-field electroretinography

Twenty-one P30 animals from three cohorts (N = 6, 7, and 8 animals) were subjected to electroretinography (ERG) recording 7 days following the HI procedure induction (P30D7) that was repeated on D14, D21, D45, and D65. The first time point (D7) was chosen because (1) the animals had to be transferred to a different animal facility for ERG recording after the induction of the HI procedure, (2) animals were allowed to recover from the HI procedure for transfer before ERG recording. Since the first ERG recoding was done at D7 (1 week after HI induction), we then decided to perform additional ERG recoding weekly, twice (D14 and D21), followed by approximately triweekly, twice (D45 and D65). Collectively, these consecutive five-time points were chosen to help determine whether the visual functional integrity recovers or deteriorates with time after HI exposure. Mice were housed in a room with controlled temperature, humidity, and light–dark cycle and were dark-adapted overnight before ERG recording. Under dim-red illumination, animals were anesthetized using an intraperitoneal injection of ketamine (80 mg/kg) and xylazine (16 mg/kg). For local anesthesia, a drop of 0.5% proparacaine hydrochloride was topically applied, and the pupil was dilated with an application of a drop of 1% tropicamide. While under anesthesia, animals were kept on a heating pad (37 °C) to prevent hypothermia. Corneal full-field flash ERG was recorded from mouse eyes using Espion system (Diagnosys LLC, MA) in accordance with the standards of International Society for Clinical Electrophysiology of Vision (ISCEV) (Doc Ophthalmol (2015) 130:1–12) adapted for mice. A drop of sterile 2.5% hypromellose ophthalmic solution (Goniovisc, HUB pharmaceuticals LLC, CA) was applied to the cornea of the dilated eyes to prevent desiccation and dehydration of the eye, and for electrical contact with the recording electrode. The reference needle electrode was inserted through the cheek, and the ground electrode was subcutaneously inserted near the base of the tail. ERG was recorded using Espion system colordome Ganzfeld for uniform illumination. Full-field ERG recording was achieved by exposing both eyes (left eye HI injured; right eye, control) simultaneously to increasing flash intensities (0.03–30 cd s/m^2^) for 400 ms. An interval 60 s was maintained between two different flash intensities. At lower flash intensities (0.03, 0.1, 0.3, 1 and 3 cd s/m^2^) 10 flashes were presented and averaged, with an interval of 4 s between the flashes and at higher flash intensities (10 and 30 cd m^2^), 4 flashes were presented and averaged, with an interval of 10 s between flashes.

Analyses of the data were carried out using Espion software (Diagnosys LLC, MA) and analyzed using Origin2018b (OriginLab Corp., MA). For quantification, the a-wave amplitude was measured from baseline to the trough of the negative deflection of the response; the b-wave amplitude was measured from the negative trough to the maximum positive peak of the response. For the acquisition of c-wave, the eyes were stimulated with light flashes of 25 cd s/m^2^ intensity for 4 s. The amplitude of the c-wave was measured from the trough of the b-wave to the next positive peak. The stimulus–response exponential fit of the dark-adapted b-wave amplitude was derived using standard Naka–Rushton function:

$$ R = R_{{\it max}} \left( {\frac{{I^{n} }}{{I^{n} + K^{n} }}} \right) $$

where R is the response amplitude at stimulus intensity (*I*), $R_{{\it max}}$ is the maximum response amplitude, K is the stimulus intensity (I) that produces a response amplitude that is half of $R_{{\it max}}$, and n is proportional to the slope of the curve at the point where the contrast is taken to be K^55^. The $R_{{\it max}}$, K, and n parameters were determined using commercial software OriginPro 2020 (OriginLab Corp., Northampton, MA).

### The grading of retinal damage

Exposure to HI conditions can result in mild, moderate, or severe injuries in human, rat, and mouse brain^56–58^ and mouse and rat retina^54,59^. While the inner retina (responsible for b-wave) is especially susceptible to various kinds of ischemic conditions, the outer retina (responsible for a-wave) is not as susceptible^54,59^. The ratio values of the b-wave/a-wave amplitudes (b/a ratio) from injured eyes were used to categorize retinal damage into mild, moderate, or severe groups. The b/a ratio above 2 is typically considered normal, which is the case with control eyes^60^. To categorize the HI injured eyes in this study into three groups, the following cutoff values of b/a wave ratio were used. HI exposed eyes with ratios above 1.75 were considered mildly injured, between 1.35–1.75 were considered moderately injured, and eyes with ratios below 1.35 were considered severely injured.

### Optical coherence tomography (OCT)

#### Acquisition

OCT images of the retinal microarchitecture were obtained using the Spectralis HRA + OCT system (Heidelberg Engineering Inc., Heidelberg, Germany). Animals were anesthetized with xylazine (10 mg/kg, Akorn) and ketamine (100 mg/kg, Akorn, Lake Forest, IL). Pupils were dilated using 0.5% tropicamide (Akorn), and contact lens was used to prevent corneal dehydration. While anesthetized, animals were kept on a heating pad (37 °C) to prevent hypothermia. OCT volume scans (30° × 25° degrees with 61 individual b-scans, 120 µm distance between B-scans) were obtained, centered on the optic nerve head of each eye (left eye, HI injured; right eye, control) using the instrument’s automatic real-time tracking mode (ART) averaging ten frames per b-scan.

#### Segmentation

Automated segmentation of the retina layers was performed using the manufacturer’s proprietary Eye Explorer algorithm (Heidelberg Engineering Inc) with manual correction, as necessary. Targeted retinal layers were retinal nerve fiber layer (RNFL), ganglion cell layer (GCL), inner plexiform layer (IPL), inner nuclear layer (INL), outer plexiform layer (OPL), outer nuclear layer (ONL), the external limiting membrane (ELM), inner segment and outer segment of the photoreceptors (IS/OS), retinal pigment epithelium (RPE), basement membrane (BM) and the choroid. (Fig. 2A). For the purposes of our studies, we recorded the average thicknesses of the following layers: whole retina, GCL, IPL, INL, OPL, ONL, and RPE.

### Immunofluorescence staining of retinal wholemounts

Immunofluorescence staining of retinal wholemounts was performed as we previously described with little modification^54,61^. Eyes were enucleated immediately post-mortem following euthanasia and fixed in 4% PFA for 10 min at room temperature, washed three times in PBS, and then transferred to methanol and kept at − 20 °C until stained. On the day of staining, eyes were rehydrated in PBS for 1 h on a rocker at room temperature. Retinas were dissected in PBS and then washed in PBS three times, 10 min each, and incubated in blocking solution (3% protease-free bovine serum albumin (BSA), and 0.3% Triton X-100 in PBS) for 1 h. Retinas were then incubated with rabbit anti-collagen IV (Millipore, AB756P) (1/500) and Guinea pig anti-GFAP (Synaptic Systems, 173004) (1/500) in the blocking solution at 4 °C overnight. Biotinylated Griffonia Simplicifolia Lectin I (GSL I) isolectin B4 (Vector Laboratories, B-1205) (1/50) was also used to stain the vasculature in the blocking solution at 4 °C overnight. Following incubation, retinas were washed three times with PBS, 10 min each, incubated with fluorescently conjugated secondary antibody, diluted (1/500), for 5 h at room temperature (RT), washed four times with PBS, 30 min each, and mounted with inner retina uppermost on a slide with DAPI Fluoromount-G (Southern Biotech). Both secondary antibodies were obtained from Jackson ImmunoResearch Laboratories, including donkey anti-rabbit-Cy3 (cat. No. 711-165-152), donkey anti-guinea pig-Alexa Fluor 488 (cat. No. 706-545-148). Streptavidin-Alexa Fluor 647 was obtained from ThermoFisher (ThermoFisher; cat. No. 016-600-084). Retinas were viewed by fluorescence microscopy, and images were captured in digital format using Nikon confocal microscope system A1+. Captured images were analyzed using NIS elements viewer (Nikon). Images from the right eye served as control for the left eye (HI injured eye).

### Trypsin-digested retinal vessel preparations

Eyes enucleated immediately post-euthanasia with carbon dioxide were fixed in 4% PFA in 0.1 M PBS for a minimum of 1 week. The cornea and lens were removed, and the whole retina was obtained under the dissecting microscope and rinsed three times in PBS, twice in distilled water, and then washed in fresh distilled water overnight at room temperature. The next day, distilled water was aspirated, and the retinas incubated in 3% trypsin (Trypsin 1:250, Difco) prepared in pH 7.8 containing 0.2 M NaF, 0.1 M maleic acid, 0.1 M Tris, at 37 °C overnight. The next morning, the whole retina was retrieved and was beaten by a thick hairbrush to loosen and separate away the nonvascular cells from the vasculature. Clean retinal blood vessels were then radially cut proximal to the optic nerve four times and then flat-mounted on glass slides for hematoxylin and periodic acid-Schiff (PAS) staining. Morphology of the nucleus was used to distinguish endothelial cells from pericytes. The endothelial cell nucleus is elongated or oval and positioned within the vessel wall along the axis of the capillary, while the pericyte nucleus is spherical, small but stain densely, and generally have a bulgy position on the capillary wall. The intact retinal wholemounts with high-quality staining were coded and subsequently used for counting. One sampling image (0.273 mm^2^) was captured from the periphery of each of the four quadrants of the retina. Thus, the total area of 0.273 × 4 = 1.09 mm^2^ per retina was used for counting. Locations where the vasculature folded on itself on the slide were not used for counting. Only retinal capillaries, but not large blood vessels, were included in the cell counts. To determine acellular capillaries, trypsin-digested retinal vessels were prepared as described above. Acellular capillaries were counted in the same field areas used to count the endothelial cells and the pericytes. Acellular capillaries were defined as collapsed capillary-sized tubes but without any nuclei along their length. After the cells and acellular capillaries were quantified, all four images were averaged to determine the mean number of cells and vascular density in the retina. Data from the right eye served as control to the HI injured left eye.

### Statistical analysis

Each experimental group (mild, moderate, and severe HI group) was compared to the control group using the Student's unpaired *t* test (two-tailed). A p-value < 0.05 was considered significant, with *p < 0.05, **p < 0.01, ***p < 0.001, and ****p < 0.0001. For a single data point, mean ± standard deviation is reported for each condition. Statistics were calculated using GraphPad Prism version 8 (GraphPad Software, La Jolla, CA).

## Results

### Retinal function is impaired at variable levels in juvenile mice subjected to hypoxia–ischemia

Juvenile C57BL/6J mice, postnatal day 30 (P30), were exposed to hypoxia–ischemia conditions according to the Rice–Vannucci model^57,62^. The left, but not the right, common carotid artery was occluded and after 2 h the animals were exposed to hypoxic conditions (10% oxygen) for 50 min. The resulting injury was limited to the left eye. The right eye from these animals was used as control.

**Figure 1:**
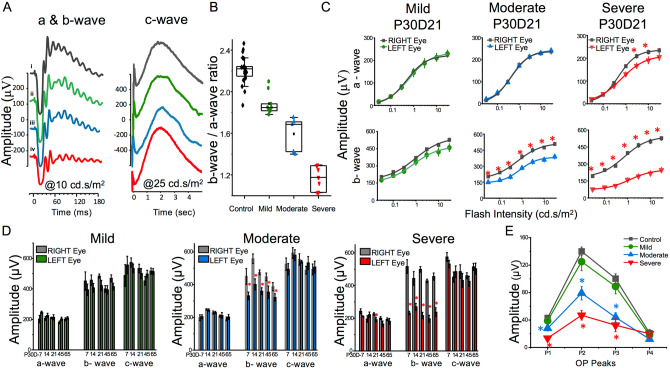
Abnormal dark-adapted electroretinogram (ERG) responses in juvenile mice after exposure to HI. (**A**) Representative ERG waveforms obtained from light flashes of 10 cd s/m^2^ (a- and b-waves, left panel) and 25 cd s/m^2^ intensities (c-waves, right panel) representing a control (right) eye (black) and HI exposed (left) eyes with different waveform profiles. At 10 cd s/m^2^; some eyes were almost completely normal (green), some other eyes showed a slight reduction in b-wave amplitude (blue) and a third group of eyes showed a substantial reduction in both a- and b-wave amplitudes (red). The c-waves of the same representative HI exposed eyes as in left panel were unaffected. (**B**) Shows the average plot of b/a ratio of control eye group and the three groups (mild, moderate, or severe groups) of HI injured eyes. (**C**) Shows amplitude (µV) of a-waves and b-waves in response to a series of intensities of single light flashes in control eyes, and in eyes with mild, moderate, and severe P30D21 HI injuries. Solid line is the Naka Rushton exponential fit of the data as an intensity-response function. (**D**) Shows summary results for amplitudes of a-wave, b-wave and c-wave obtained from control eyes, and eyes with mild, moderate, and severe HI at P30D7, P30D14, P30D21, P30D45 and P30D65. Peak amplitudes were collected at flash intensity 10 cd s/m^2^ for a-wave and b-wave and 25 cd s/m^2^ for c-wave. (**E**) shows amplitude responses of the four major oscillatory potential (OP) peaks of control eyes, and from eyes with mild, moderate, and severe HI exposure. Data from 21 animals from three cohorts (N = 6, 7, 8 animals) were analyzed and presented as mean ± SD.

Electroretinography (ERG) was used to assess the functional integrity of the retina in HI exposed juvenile mice. After HI exposure at P30, 21 mice from 3 cohorts were assessed by ERG on post-HI day 7 (D7) (P30D7), and at timed intervals thereafter, designated P30D14, P30D21, P30D45 and P30D65. ERG was performed on dark-adapted mice and eyes were subjected to light flashes of sequentially increasing intensities. Figure 1A shows representative ERG a- and b-waves generated using 10 cd s/m^2^ flashes, and the c-wave generated using 25 cd s/m^2^ flash intensities and recorded over 4 s. Compared with the control eye response (black traces), either there was no reduction in a- or b-wave forms (green traces); moderate reduction in the b-wave only (blue traces), or substantial functional impairment with mild reduction in a-wave and substantial reduction in b-wave (red traces) observed in the HI exposed eyes. The c-wave appeared normal for all HI exposed eyes (Fig. 1A; right panel). The inner retina, responsible for the ERG b-wave, is especially susceptible to various kinds of ischemic conditions, while the outer retina, responsible for the ERG a-wave, is less susceptible to HI^54,59^. Thus, the ratio of b-wave/a-wave amplitudes (b/a ratio) from P30D21 injured eyes were used to categorize retinal damage into mild, moderate, or severe groups^60^. The b/a ratio above 2 is typically considered normal, which was the case with control eyes (Fig. 1B; black). To categorize the HI injured eyes in this study into three groups, the following cutoff values of b/a wave ratio were used. HI exposed eyes with ratios above 1.75 were considered mildly injured (Fig. 1B; green), between 1.35–1.75 were considered moderately injured (Fig. 1B; blue), and eyes with ratios below 1.35 were considered severely injured (Fig. 1B; red). These differences in severity of injury are well documented responses of the central nervous system, including the retina and brain, in different individuals after HI exposure in both humans and preclinical Rice–Vannucci rodent model^58,59,63,64^.

The four main components of an ERG response from each eye were further analyzed. The peak amplitudes of the a-wave derived from photoreceptors, the b-wave derived from the inner nuclear layer; mainly bipolar cells, the oscillatory potentials, OP; potentially derived from the feedback pathways among ganglion cells, amacrine cells and bipolar cells, and the c-wave derived from retinal pigment epithelium (RPE). Figure 1C shows a-wave and b-wave amplitudes (µV) as a function of flash intensities in mild, moderate, and severe P30D21 HI injury. As compared to the control eyes, the a-wave amplitude (Fig. 1C; upper panels) remained unperturbed in eyes with mild (green curve) and moderate (blue curve) injury, while eyes with severe injury (red curve) showed reduced a-wave amplitudes only at higher flash intensities between 1 to 10 cd s/m^2^. For b-wave comparisons (Fig. 1C; lower panels), mildly injured eyes showed slightly reduced amplitudes (green curve). In contrast, eyes with moderate (blue curve) and severe (red curve) injuries showed significantly lower b-wave amplitudes.

To more comprehensively examine retinal function, we used Naka–Rushton equation fitting^55^. Utilizing different parameters, the Naka–Rushton equation quantifies selective changes in b-wave responses to a range of light intensities. Values of different Naka–Rushton equation parameters, R_max,_ *K*_,_ and n, are shown in Table 1. R_max_ reflects both the cell number and the increment (μV/quanta) related to each b-wave generating cell. The HI injured eyes showed reduced R_max_ values that correspond with the severity of the injury. *K* represent the sensitivity responses of the retina to flash stimulus; an increase in *K* value means a stronger flash stimulus is needed to generate b-wave of similar amplitude. *K* values of the mildly injured group exhibited slight enhancement in sensitivity response as compared to their control eyes. In contrast, the moderately injured eyes showed a moderate reduction in sensitivity, and the severely injured eyes showed a dramatic reduction in light sensitivity as compared to their corresponding control eyes (Table 1). Together, results of the b-wave amplitude fitting to Naka–Rushton equation further confirmed our categorization, whereby the three HI injury groups showed distinct values for the different parameters of the Naka–Rushton equation.

**Table 1:** Naka–Rushton parameters (means ± SD) for dark-adapted b-wave amplitudes of P30D21 control and HI injured eyes.

Naka–Rushton fit parameters b-wave	Mild (N = 9)		Moderate (N = 5)		Severe (N = 7)	
	Control	HI	Control	HI	Control	HI
R_max_ (μV)	550.096 ± 65.6	485.52 ± 41.9	517.69 ± 43.2	385.62 ± 26.4	543.11 ± 104.6	250.78 ± 56.7
K (cd s/m^2^)	0.92 ± 0.48	0.76 ± 0.33	0.63 ± 0.33	0.8 ± 0.3	0.71 ± 0.62	1.23 ± 1.17
*n*	0.72 ± 0.31	0.93 ± 0.4	0.8 ± 0.34	1.08 ± 0.46	0.83 ± 0.70	0.86 ± 0.65

*R*_*max*_ is the maximum response amplitude to a range of flash intensities (I)s given in cd s/m^2^. *K* is the stimulus intensity (*I*) that produces a response amplitude that is half of *R*_*max*_. *n* is proportional to the slope of the curve at the point where the contrast is taken to be *K.*

Amplitudes of a-wave and b-wave collected at additional time points (P30D7, P30D14, P30D45, and P30D65) were similar to those obtained for P30D21 (Fig. 1D). C-wave amplitudes appeared to be less affected over time by HI conditions (Fig. 1D). Amplitude values for the four OPs were calculated and summarized in Fig. 1E. Both control eyes, and mild HI injury eyes had similar OP amplitudes (Fig. 1E; black vs. green plots). In contrast, the eyes with moderate (Fig. 1E; blue) and severe (Fig. 1E; red) injuries showed significantly reduced OP1, OP2, and OP3 amplitudes compared with control eyes. OP4 amplitude was not affected by HI injury relative to the control eyes, and all eyes with varying degrees of HI injury had similar OP4 amplitudes. Taken together, our ERG data demonstrated that exposure of the juvenile mice to HI conditions irreversibly compromised the functional integrity of retinal neurons, especially in the inner retina.

### Retinal layers show thinning in juvenile mice subjected to hypoxia–ischemia

**Figure 2:**
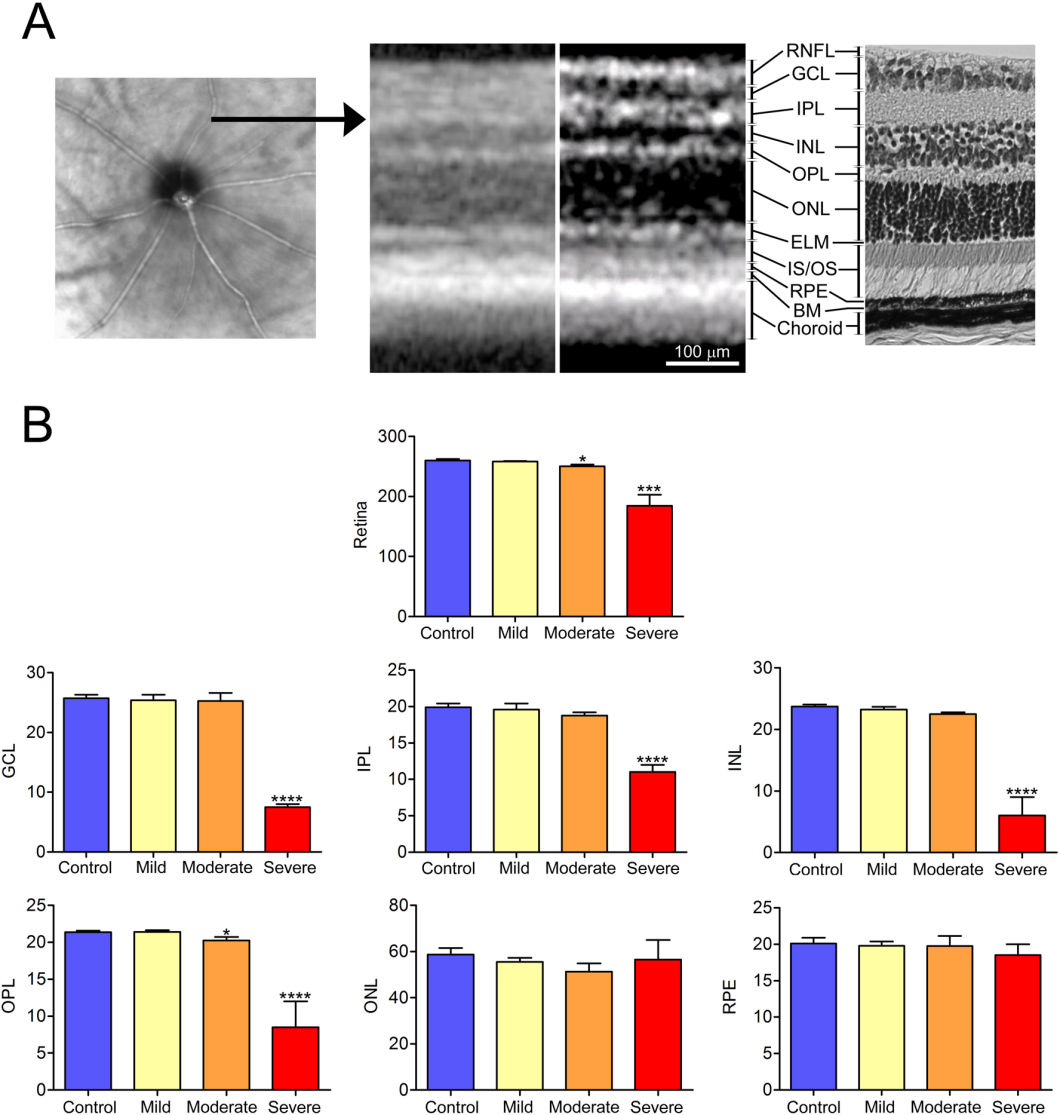
Retinal thinning with preservation of outer retinal layers in the juvenile mouse after exposure to HI conditions. (**A**) Original OCT image from control retina (left) with the horizontal black arrow on the superior side of the retina, pointing to the location of the cross-sectional OCT image (second from the left). The same cross-sectional OCT image was color-contrast modified and presented next to a photomicrograph of normal mouse retina. Eleven retinal layers were identified and marked besides the two images. (**B**) Automated segmentation was used to measure the full thickness of the retina along with GCL, IPL, INL, OPL, ONL and RPE layers using a proprietary software algorithm. The measurements were taken from scans acquired from the superior retina of right (control) and left (HI injured) eyes of 11 animals revealed evidence of significant retinal thinning with preservation of outer retinal layers in eyes with moderate and severe HI injury. Data presented as mean ± SD. *p < 0.05, ***p < 0.001, ****p < 0.0001. Scale bar = 100 μm. *RNFL* retinal nerve fiber layer, *GCL* ganglion cell layer, *IPL* inner plexiform layer, *INL* inner nuclear layer, *OPL* outer plexiform layer, *ONL* outer nuclear layer, *ELM* the external limiting membrane, *IS/OS* inner segment and outer segment of the photoreceptors, *RPE* retinal pigment epithelium, *BM* basement membrane; and the choroid.

To examine the effect of HI on the morphology of the retinal layers, we used optical coherence tomography (OCT) to image the eyes in vivo. OCT is widely used to diagnose many eye diseases, including various ischemic eye conditions, including diabetic retinopathy, glaucoma, and macular degeneration. Because ERG studies showed that the HI induced damage is irreversible, we reasoned that assessing the integrity of the retinal structure using OCT at a single time point is sufficient. Eleven additional P30 mice were subjected to HI conditions, and OCT was performed 90 days afterward (P30D90). The OCT images allowed for segmentation of eleven major layers of the retina identifiable in hematoxylin and eosin (H&E) stained adult mouse retina (Fig. 2A). For the purposes of this study, full retinal thickness along with the thickness of ganglion cell layer (GCL), inner plexiform layer (IPL), inner nuclear layer (INL), outer plexiform layer (OPL), outer nuclear layer (ONL), and retinal pigment epithelium (RPE) layer were measured in scans acquired from retina superior to the optic nerve head of the right (control) and the left (injured) HI eyes of each animal. Animals with mild, moderate, and severe injuries were identified here based on whole retinal thickness. The mild HI group showed similar thickness to control eyes. In contrast, both the moderate and the severe HI groups demonstrated significantly different retinal thicknesses from control eyes. The severe HI group displayed significant thickness differences in GCL, IPL, and INL. The OPL was significantly thinner in both moderate and severe HI groups, but not in the mild HI group. No significant differences between eyes and groups were observed in either the ONL or RPE thickness for the three HI groups (Fig. 2B).

### Development of astrogliosis in the retinas of juvenile mice subjected to hypoxia–ischemia

**Figure 3:**
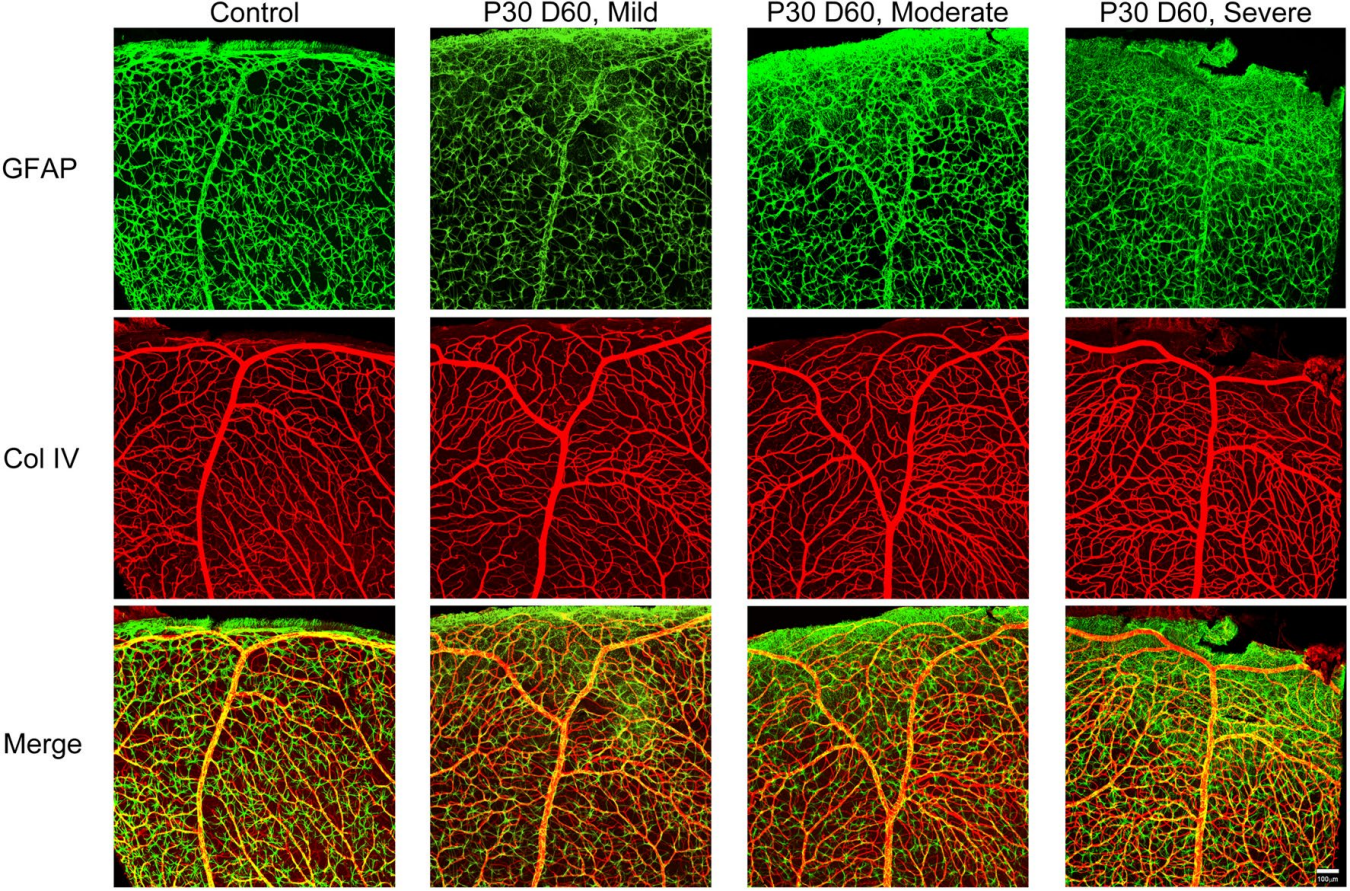
Glial activation in juvenile mouse retina after exposure to HI. Representative images were taken from the periphery of the superior retina of control (right eye), mild, moderate, and severe HI injured (left) eyes (P30D60). Wholemounts were stained for GFAP and Col IV; GFAP is an intermediate filament protein expressed in astrocytes and activated Müller cells. Control retina showed normal astrocyte morphology: stellate-shaped, thin cytoplasmic processes, limited processes intermingling and no scar formation. Astrocytes in mild HI retina were like those in the control retina. Müller cells, however, show some reactivity as shown by sand-like GFAP staining. Astrocytes in moderate and severe HI retinas are reactive, especially at the periphery edge of the retina. Retinas from at least four mice per group were examined. Scale bar, 50 µm.

Astrocytes reside in the retinal GCL and nerve fiber layer. They interact with the retinal ganglion cells and blood vessels in the layer^65^. Müller cells span the retina from inner to outer limiting membranes with cell bodies that reside in the inner nuclear layer of the retina. Both astrocytes and Müller cells are referred to as macroglia or glial cells of the retina. Glial cells play crucial roles in homeostasis and function of neurons and blood vessels^66^. Under ischemic conditions in the central nervous system, glial cells become reactive. Cardinal feature of glial reactivity are the upregulation of glial fibrillary acidic protein (GFAP) and changes in the morphology of glial somas and processes^67,68^. To study the reactivity of the glial cells in the retina, GFAP expression was examined in immunolabeled wholemount retinas from control (right eye) and HI injured (left eyes), P30D65 animals. Representative images from the superior periphery of a control retina, and retinas with mild, moderate, and severe HI injury are shown in Fig. 3. Astrocytes in the control retina displayed a stellate shape with distinct soma and thin cytoplasmic processes, with a morphology typical of normal retinal astrocytes. The astrocytes in the control retina tended to touch each other but without any bundling or forming scars. Furthermore, no detectable GFAP expression was observed in Müller cells. With mild HI, retinal astrocyte morphology grossly appeared similar to their control counterparts. However, Müller cells at the retinal periphery edge showed reactivity, as GFAP expression was detectable in their trunks and end-feet; the trunk normally extends throughout the inner retina until the end-feet reach the ganglion cell/nerve fiber layer and inner limiting membrane (Fig. 3). In the moderately and severely affected retinas, astrocytes showed features typical of reactivity. They lost their stellate shape; cytoplasmic processes became thicker and tended to bridge together with processes from other cells and formed scars at the retinal periphery (Fig. 3). Furthermore, Müller cells appeared reactive with enhanced GFAP expression.

### HI exposure results in retinal vascular degeneration in juvenile mice

**Figure 4:**
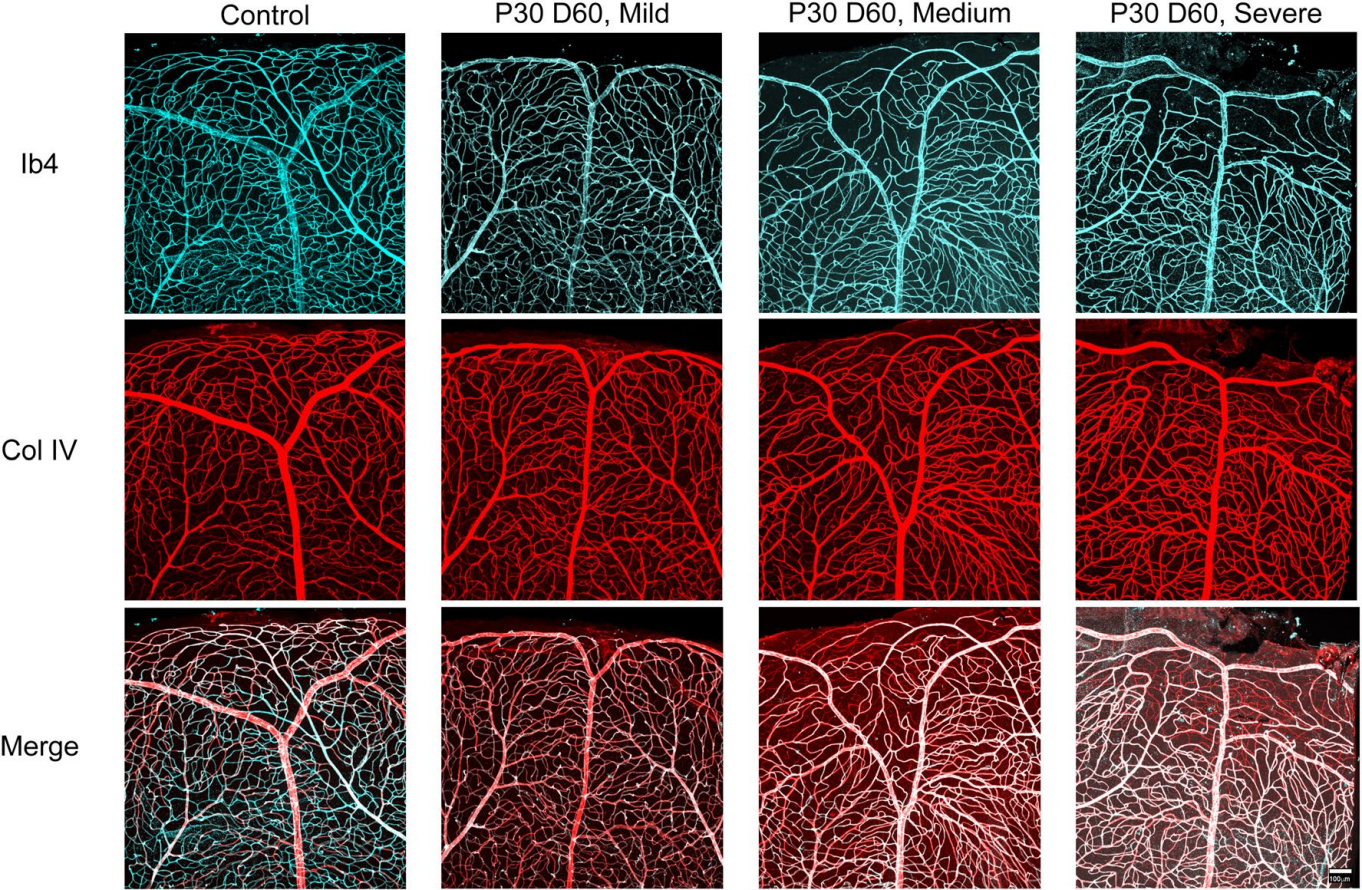
Vascular degeneration in juvenile mouse retina after exposure to HI. Representative images from the periphery of the superior retinas of control (right), mild, moderate, and severe HI injured (left) eyes from P30D60 mice. Retinal vasculature was labelled with Griffonia simplicifolia isolectin B4 (IB4, cyan) and anti-collagen IV antibody (Col IV, red). Vascular damage is obvious in images representing the moderate and severe HI groups. Retinas from at least four mice per group were examined. Scale bar, 50 µm.

To investigate the effect of HI on the integrity of blood vessels in the retina of juvenile mice, wholemount retinas from control (right eye), mild, moderate, and severe HI (left eyes) of P30D65 mice were immunolabelled with anti-collagen IV antibody and stained for isolectin B4. Collagen IV is expressed in basement membrane of blood vessels regardless of whether the vessel is perfused (live vascular cells) or not (loss of vascular cells). Isolectin B4 labels perfused blood vessels with viable vascular cells. Representative images of blood vessels in the superior retinas of control and HI injured eyes are shown in Fig. 4. Retinas from control eyes displayed normal vascular structure. The blood vessels in mildly HI injured retinas had a similar vascular structure to those in the control eyes. In contrast, retinas of both moderately and severely HI injured eyes showed a significant vascular degeneration, especially at the periphery. This damage resulted in fewer blood vessels with viable vascular cells (co-labeled for collagen IV and isolectin B4) and an abundance of acellular capillaries (staining only with anti-collagen IV) (Fig. 4).

**Figure 5:**
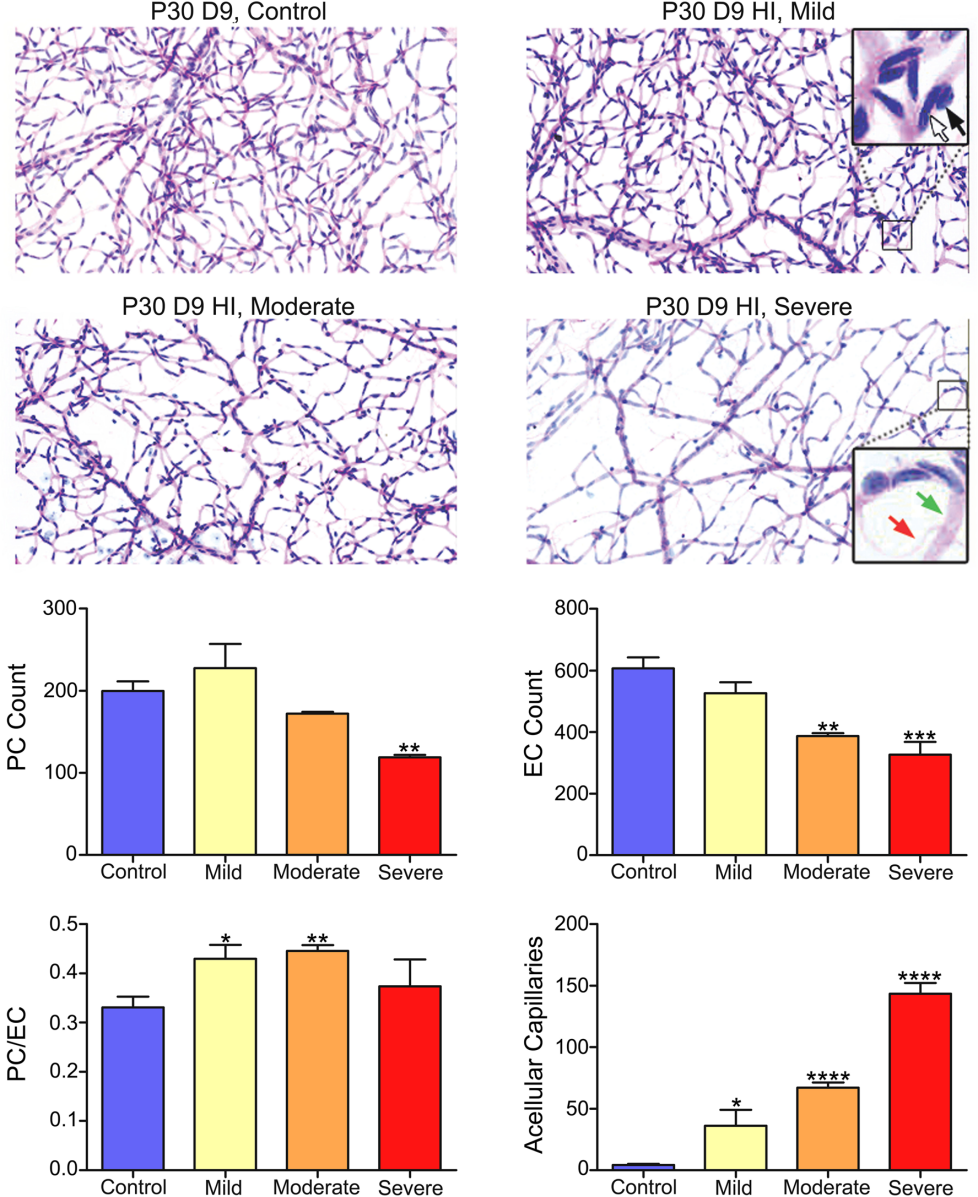
Vascular degeneration in juvenile mouse retina after exposure to HI as shown by trypsin digest preparations. Representative images were captured from the peripheral retinal vasculature of control (right), mild, moderate, and severe HI injured (left) eyes from P30D9 mice. The endothelial cells nuclei reside within the vessel wall along the capillary wall (white arrow); Nuclei of pericytes generally land on the outside of the capillary wall (black arrow). Number of pericytes, endothelial cells, the pericyte/endothelial (PC/EC) ratios, and the acellular capillaries (red arrow) are summarized. Data from 5 control eyes, 3 mildly injured eyes, 3 moderately injured eyes and 2 severely injured eyes were analyzed and presented herein. Data presented as mean ± SD. Preparations were imaged using the Aperio slide scanner and × 20 objective was used.

To quantify the effects of HI on retinal vasculature integrity and endothelial cell and pericyte numbers, trypsin digests were prepared from retinas of control eyes and eyes with mild, moderate, and severe P30D9 HI injury (Fig. 5). Grossly, unlike retinas from animals with mild HI injury, retinas with moderate and severe HI injury in P30D9 animals had lower vascular density as compared with retinas from the control eyes. The numbers of pericytes, endothelial cells, and acellular capillaries and the pericyte/endothelial (PC/EC) ratios were quantified (summarized in Fig. 5). The number of pericytes was significantly lower in animals with severe HI. The number of endothelial cells was lower in both the moderately and severely HI injured retinas. Pericyte/endothelial cell (PC/EC) ratios were significantly higher in mild and moderate injury HI groups because EC numbers were reduced more than the PC numbers in these two groups when compared with the control group. PC/EC ratio was not different in the severe HI group because both the EC and PC equally decreased in this group when compared with the control group. The number of degenerated capillaries was significantly higher in all three HI groups as compared to the control group. Thus, HI caused retinal vascular degeneration in juvenile mice, which varied in magnitude and was consistent with the severity of the retinal injury as determined by the reduced retinal function.

## Discussion

Here we determined that HI in juvenile mice disrupts retinal neurovascular integrity and function. Our data demonstrated interindividual variability in retinal injury after exposure to HI. This is in line with previous reports that demonstrated exposure to HI conditions resulted in various levels of injuries (mild, moderate and severe) in different individuals including human, rat and mouse neonatal retinas^54,59,63,69,70^ and brains^56–58,64^. The exact reasons for these interindividual variabilities in HI-induced injuries are not completely understood. Age may play a role as the magnitude of severe and moderate forms of the retinal vascular injury after exposure to HI are more in neonatal mice^54^ as compared to juvenile mice (this report). Hemodynamic variations may affect the severity of HI-induced injury^71^ as different retinas have unique vascular structures. Sex of the animal may also contribute to the severity of injury of the retina as in the brain after exposure to HI conditions^72,73^. Although this was not directly addressed here, the impact of sex on the severity of retinal injury deserves a more careful evaluation and is a subject of future investigation.

We noted retinal macroglia reactivity and retinal capillary degeneration in moderate and severe HI groups, especially in the peripheral retina. Functional and structural studies using ERG and OCT analysis in vivo demonstrated preservation of the outer retinal structure and function including the a-wave, which reflects the activity of photoreceptors^74^. The c-wave, which derives from RPE cells^75^ and depends on photoreceptors integrity was normal in all animals^76^. Together, these results suggest that HI does not affect photoreceptor and RPE physiology, or their interactions, in juvenile mice. The OCT results corroborated these findings, as both the OPL and RPE layers were preserved in all examined animals. These studies will further benefit by carefully evaluating the expression and localization of synaptic markers like PSD95, Gephyrin, Ctbp2, mGluR6 and VGlut1 in future studies.

Both ERG and OCT studies supported inner retinal damage in animals subjected to HI. Both moderate and severe HI groups displayed a reduced ERG b-wave amplitude and a shift in light sensitivity. The ERG b-wave response mainly reflects the electrical activity of bipolar cells^77^, an activity that initiates at its dendrites in the OPL. In addition, pharmacological and genetic mouse studies showed that horizontal cells^78^ and the third-order neurons, amacrine cells, and ganglion cells^79–81^ contribute to the b-wave response. Therefore, a loss in amplitude and reduction of light sensitivity indicates degeneration of inner retina cells. Three of the four ERG oscillatory potentials were also attenuated in moderate and severe HI groups. Oscillatory potentials reflect the inhibitory feedback activity among amacrine cells, bipolar cells, and ganglion cells^82^. The intermediate OP responses which we found to be severely reduced following HI insult in mice, are generated by action-potential-independent interactions between third-order neurons in the ON pathway of the rabbit retina^83^. Thus, exposure to HI leads to the impaired neuronal activity of all neurons in the inner retina. This is supported by the OCT data, which showed a significant reduction in the overall thickness of the whole retina, and specifically the OPL, in moderate and severe HI groups. The OPL layer harbors synapses between photoreceptors and the bipolar cell second-order retinal neurons.

The amplitude of b-wave can be significantly reduced under severe or even subtle ischemic conditions in humans and mice as a result of blocking the central retinal artery^84–86^. The amplitudes of OPs are responsive to even subtle retinal ischemia when other ERG components remain unchanged, as recognized in diabetic retinopathy^87–89^. Furthermore, several mouse knockouts for genes critical for normal vascular development and homeostasis in the retina (e.g., frizzled-4, and Lrp5) result in compromised b-wave and OP amplitudes^90,91^. Our findings are consistent with these reports that further underscore the sensitivity of retinal neurons, specifically those in the inner retina to ischemia. Neuronal degeneration following HI is postulated to result from glutamate excitotoxicity, free oxygen radical accumulation, inflammation, and disruption of the blood-retinal barrier^51^.

Here we observed chronic macroglia activation in the retinas of mice with moderate and severe HI injury. Astrogliosis is noted under ischemic conditions in the central nervous system, including the retina. Prolonged astrogliosis can compromise blood-retinal barrier integrity, which in turn contributes to neurodegeneration^92,93^. Thus, the reactivity of both astrocytes and Müller cells may contribute to the neurovascular degeneration in retinas with HI injury.

In conclusion, this report demonstrates that both neurons and blood vessels in the inner retina of juvenile mice are susceptible to damage by HI. As in the case of ischemic stroke in the pediatric population, our studies showed that the severity of the injury resulting from HI varied among individual mice. Our data suggest that ischemic stroke in pediatric patients (children from 29 days to 18 years old) is very likely to cause retinal damage in addition to brain damage, and therefore warrants clinical follow up to identify and manage potentially debilitating ocular pathology.
